# Impact of Increased Economic Burden Due to Human Echinococcosis in an Underdeveloped Rural Community of the People's Republic of China

**DOI:** 10.1371/journal.pntd.0000801

**Published:** 2010-09-14

**Authors:** Yu Rong Yang, Gail M. Williams, Philip S. Craig, Donald P. McManus

**Affiliations:** 1 Ningxia Medical University, Yinchuan, Ningxia Hui Autonomous Region, People's Republic of China; 2 Molecular Parasitology Laboratory, Queensland Institute of Medical Research, Brisbane, Australia; 3 School of Population Health, University of Queensland, Brisbane, Australia; 4 Biomedical Sciences Research Institute, School of Environment and Life Sciences, University of Salford, Salford, United Kingdom; Universidad Peruana Cayetano Heredia, Peru

## Abstract

**Background:**

Ningxia is located in western People's Republic of China, which is hyperendemic for human cystic echinococcosis (CE) throughout the entire area with alveolar echinococcosis (AE) hyperendemic in the south. This is in part due to its underdeveloped economy. Despite the recent rapid growth in P.R. China's economy, medical expenditure for hospitalization of echinococcosis cases has become one of the major poverty generators in rural Ningxia, resulting in a significant social problem.

**Methodology/Principal Findings:**

We reviewed the 2000 inpatient records with liver CE in surgical departments of hospitals from north, central and south Ningxia for the period 1996–2002. We carried out an analysis of health care expenditure of inpatient treatment in public hospitals, and examined the financial inequalities relating to human echinococcosis and the variation in per capita income between various socioeconomic groups with different levels of gross domestic product for different years. Hospital charges for Yinchuan, NHAR's capital city in the north, increased approximately 35-fold more than the annual income of rural farmers with the result that they preferred to seek health care in local county hospitals, despite higher quality and more efficient treatment and diagnosis available in the city. Household income levels thus strongly influenced the choice of health care provider and the additional expense impeded access of poor people to better quality treatment.

**Conclusions/Significance:**

Information on socioeconomic problems arising from echinococcosis, which adds considerably to the burden on patient families and communities, needs to be collected as a prerequisite for developing policies to tackle the disease in rural Ningxia.

## Author Summary

This paper compares medical expenditure for hospital treatment of echinococcosis in NHAR, western People's Republic of China, for different years, different regions and different socioeconomic groups. The results show that the level of household income strongly influences health care decisions. This study represents an effort to determine the effect of hospital charges for inpatient treatment of echinococcosis on the choice of provider in NHAR, and quantitatively examines this topic for the rural poor. The findings show that low income individuals from rural areas opted to visit a local county hospital rather than an urban hospital for hydatid surgery despite the inferior infrastructure, personnel and general health care facilities available. There are a number of policy implications. For example, enhancing the quality and service of county hospitals in rural areas will benefit those with lower incomes, thus improving access of rural residents to health facilities for higher quality diagnosis and efficient treatment. Thus, we advocate that government policy should be to increase investment in health care in poor rural areas, and to launch relevant medical aid projects to help those in poverty.

## Introduction

Since the inception of market reforms in the early 1980s, the annual health expenditure of People's Republic of China (P.R. China) has increased consistently [1], [2]. But, contrary to this increase, two national healthcare surveys [3], [4] showed that health insurance decreased from 30.2% of coverage in 1993 to 23.6% in 1998. Moreover, the rural insurance system has almost collapsed [5]–[8]. Thus, the majority of rural residents have to pay all their medical expenses personally and are therefore ‘out-of-pocket’ for any health services they require. The rapid escalation of medical costs, largely due to the frequent use of advanced medical technologies and over-prescription of drugs by health care providers [1], [9], accompanied by a lack of insurance coverage, has inevitably caused severe financial hardship for many households in P.R. China. This is particularly so for low income rural families. In this way, poor health is an important source of transient poverty.

Ningxia Hui Autonomous Region (NHAR) is an underdeveloped provincial autonomous region in western P.R. China [10]. Human cystic echinococcosis (CE) is hyperendemic throughout NHAR whereas CE and alveolar echinococcosis (AE) are co-hyper-endemic in the southern areas [11], due, in part, to poverty and poor economic development. Sheep farming is the main source of income due to the prevailing socio-religious Islamic culture and the favourable conditions for growing crops and breeding livestock [11], [12]. This article focuses on the analysis of health care expenditure from public hospital records in NHAR, and examines the health and financial inequalities relating to human echinococcosis. It also examines variation in per capita income between various socioeconomic groups and different regions with different levels of gross domestic product (GDP) for NHAR. The purpose of this study was to gain some insight into the inequality in health services and their utilization, and health care expenditure through detailing hospital charges and average personal income in NHAR, so as to inform policy development, a pre-requisite for better understanding the interactions of poor households with health systems in different contexts so as to promote equitable and universal access to basic health care.

## Methods

### Study area and economic development

**Figure 1:**
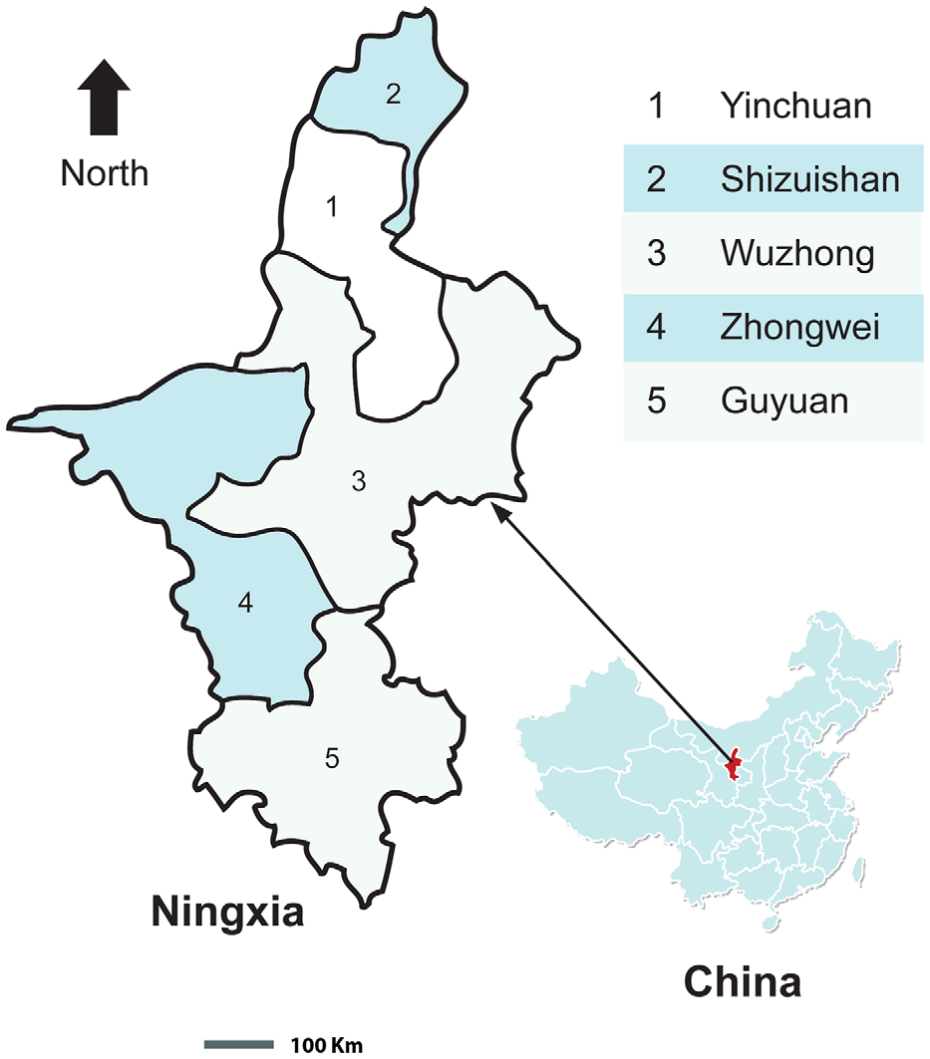
Geographic and economic zonal map of NHAR, P.R. China.

NHAR is one of five provincial level autonomous regions in P.R. China. With a total area of 66,400 Km^2^, the Hui, one of the officially recognized nationalities of P.R. China, make up 34% of the total population of 5.9 million. NHAR is located in the middle reaches of the Yellow River in northwest P.R. China, and it has five regional prefecture municipalities or cities: Yinchuan and Shizuishan in the north, central Wuzhong and Zhongwei, and Guyuan in the south (Fig. 1). NHAR is mostly deserted and is sparsely settled, but the vast plain of the Yellow River in the north has been irrigated for centuries; over the years an extensive system of canals has been built. Desert and grazing land make up most of the area. Extensive land reclamation and irrigation projects have increased cultivation. The fertile plain is ideal for the cultivation of fish and favorable agricultural conditions for growing crops and breeding livestock in north and central NHAR. This greatly benefits the northern Yellow River regions, notably Wuzhong municipality, including Qingtongxia, Wuzhong City and Zhongwei county, as well as the capital city Yinchuan, which is the economic and cultural centre, and is the most developed region in NHAR. This is in direct contrast to south NHAR (Fig. 1) which is mountainous with low production levels and poor economic development. This variability in natural environment and resources in the different geographical areas has led to economic disparity between south, central and north NHAR (see Tables 1 and 2). There are two geographical and administrative centres in NHAR: Guyuan in the south and Yinchuan in the north. The hospitals in Guyuan (The Second Provincial Hospital of NHAR) and in Yinchuan are responsible for patients from the whole of NHAR and receive financial support from both the local and central NHAR governments.

**Table 1:** A comparison of local GDP to local hospital charges between 1998 and 2002, and the average charge of total hospital costs from 1996 to 2002.

Variable	Yinchuan	Wuzhong	Guyuan	Counties with low GDP*
Average hospital charges, 2002	699	500	457	360
Average hospital charges, 1998	450	263	224	265
Ratio 2002: 1998 for average hospital charges	1.6	1.9	2.0	1.3
Ratio of per capita GDP, 2002:1998	2.3	1.5	2.0	1.3
Average hospital charges, 1996–2002	540	304	251	208
Difference between 1998–2002 by total				
OR (95% Cl)	1.5 (1.3–1.0)	1.9 (1.5–2.4)	2.0 (1.6–2.6)	1.4 (1.0–1.8)
P-value	<0.01	<0.01	<0.01	<0.05

*Mean values for Zhongwei, Tongxin and Haiyuan counties with low per capita GDP plus Xiji county with the lowest per capita GDP in south NHAR.

**Table 2:** Descriptive statistic for local entire GDP/per capita GDP levels, per capita income/deposited income compared with hospital charges by category for 2002 in NHAR (source: references [13], [17]).

Location and economic level	Per capita income (US$)	Deposited income (US$)	Hospital charges (US$)	%^1^	%^2^
Yinchuan	2200	1290	699	32	54
Wuzhong	1000	484	500	50	103
Guyuan	750	345	457	61	133
Low GDP counties #	550	245	360	65	147
			699	318	3495
Rural farmer §	220	∼20	457	208	2285
			360	163	1800

%^1^ indicates the outcome of a comparison of health care expenditure/hospital charges with per capita income;

%^2^ indicates the outcome of a comparison of health care expenditure with per capita deposited income;

#Mean values for Zhongwei, Tongxin and Haiyuan counties with low per capita GDP plus Xiji county with the lowest per capita GDP in south NHAR.

§Projected costs for a poor farmer from a rural area seeking inpatient healthcare at different public hospitals. The gap between income and hospital payment accounted for 163% of per capita income and 1800% of deposited income if the farmer sought inpatient treatment at a local county hospital; it accounted for 208% of per capita income and 2285% of deposited income if treatment was sought in Guyuan, and 318% of per capita income and 3495% of deposited income if sought in Yinchuan.

### Source of data

The data used in this study mainly involved two components. The first component comprised regional/local economic data for different years and socioeconomic groups and population demographic searches by locality; these data were compared with relevant hospital data. The second component included hospital inpatient records, particularly hospitalization charges, which were collected for all echinococcosis patients who had previously registered in a public hospital in NHAR. The full details of the data sources are described in the following sections.

### Socioeconomic data and population demographic searches

The searched data included local population demographic status (using census data); GDP per year; per capita GDP and per capita income and/or per capita deposited income by locality; and basic livelihood proportions of per capita expenditure of households in various socioeconomic population groups. Available statistical data for NHAR [13] were used to calculate the average per capita income level for different socioeconomic groups/localities by comparison with the average entire rural GDP level (Table 3), which obviously reflects the living standard levels of different socioeconomic groups. All other data used in this study were obtained from searches of locally available information cited in the references [13]–[16]. The search methods for obtaining the sourced data included electronic publications of the local NHAR literature, annual local government reports/documents and social science abstracts for NHAR.

**Table 3:** Per capita expenditure (US$) of households (basic livelihood proportions) for urban and rural areas in NHAR in 2002 (Source: reference [13]).

Expenditure item	Urban setting (Yinchuan)	Rural, upper- middle level	Rural, middle level	Rural lower- middle level	Rural lowest level
Food	308 (33.9*)	245 (47.4)	205 (50.5)	162 (53.2)	111 (55.6)
Clothing	110 (12.1)	28 (5.5)	22 (5.5)	16 (5.3)	11 (5.3)
Appliances	61 (6.7)	20 (3.9)	15 (3.6)	11 (3.6)	7 (3.6)
Medicine	73 (8.0)	34 (6.5)	24 (6.0)	18 (5.8)	11 (5.7)
Transport and communication	97 (10.7)	45 (8.7)	32 (7.8)	21 (6.9)	12 (5.9)
Education, culture and entertainment	106 (11.6)	61 (11.9)	46 (11.3)	32 (10.6)	20 (10.0)
Housing	137 (15.0)	72 (14)	53 (13.0)	39 (12.7)	24 (12.1)
Unexpected expenditure items	18 (2.0)	11 (2.1)	9 (2.3)	6 (1.9)	4 (1.8)
Total (US$)	910	516	405	305	200

*Percentage (%) value of the proportion of household expenditure on the items shown.

### Medical details and expenditure of inpatient surgery for hydatid disease

We reviewed the records of 2000 patients from 24 public hospitals in 20 administrative districts across the whole of NHAR [17]. The hospitals included public hospitals in 18 of these districts, two in Guyuan and four in Yinchuan. All patients, between 1996 and 2002, underwent surgery for removal of liver hydatid cysts in the surgical departments of these public hospitals in north, central and south NHAR (Fig. 1). Data were extracted on sex, age, occupation, nationality and domicile, duration of hospitalization, cost of each procedure for diagnosis and treatment, and cost of hospital stay.

### Financial estimates

The extent of urban-rural income inequality can be manifested by the real per capita urban-to-rural consumption ratio as shown in Table 3. Consumption is considered to be a better measure of living standards than income [18].

The costs of hospital charges for hydatid surgery mainly included general expenses (hospital stay, staff, and medications), surgical costs (surgery fee, costs for anesthetization and monitoring, and other expenditure, such as oxygen, blood transfusion costs, emergency equipment and essential drugs). Other components of the hospital charges were radiology/imaging examinations, laboratory testing, and exploratory surgery/post-surgical nursing and prescription charges.

The economic consequences of illness and costs for health care and treatment in public hospitals in various regions of NHAR were compared across different socioeconomic groups and also by their geographic location.

### Measurement of the financial burden of hospital inpatients with echinococcosis

Financial estimates were based on the actual costs of the treatment of echinococcosis cases in public hospitals in various regions in NHAR. A comparison of increased charges for hospital treatment between different years (comparing 2002 with1998) and between different regions (different locations with differing economic levels) was also carried out.

The GDP is a common measure of welfare for socioeconomic development. Generally, per capita income estimates are based on the present value of income/earnings. The deposited per capita income was chosen in this study to be a financial measurement for three reasons: (i) it is an after-tax income for the individual; (ii) it already includes both non-labour and labour income; (iii) it incorporates in-kind incomes as well.

The ‘per capita’ income and household expenditure for education, health care, food, food for livestock and purchase of chemical fertilizer for crops, were compared to estimate livelihood levels. The cash income per person increased as household income increased, as also did expenditure on education, health care and food, suggesting that using per capita income/deposited income can reflect real differences in the income levels of households (see Table 3, data of livelihood expenditure weights from the 2004 Statistics Year Book for NHAR) [13]. Therefore, the direct cost burden is a measure of the proportion of per capita income that can represent the household burden in most rural families in NHAR. In this study, the direct cost of hospitalization for echinococcosis cases was measured as the proportion of household expenditure that was spent on health care, indicating the financial burden placed on the household by the cost of seeking treatment.

### Data management and analysis

The mean, standard deviation (SD), and the percentiles were calculated for general descriptions of inpatient records, including demographic data for age, occupation, clinical information for liver CE lesion size/numbers, and data for length of hospital stay, which included length of pre- and post-surgery stay. An analysis of hospital charges comparing per capita income/per capita deposited income was carried out to demonstrate what proportion of average individual income was used for hospitalization costs. This can reflect the average economic burden in various socioeconomic groups of people. Pearson's chi-square test for independence was used in order to test differences between years, locations and people-groups. The level of statistical significance was set at P = 0.05 unless otherwise stated. All statistical analyses were performed using SPSS 13.0 software (SPSS Inc. Chicago, III, USA).

### Ethical approval

Ethical clearance for the study was given by the Ningxia Medical University Ethics Committee and The University of Queensland Ethics Committee, and sanctioned by local hospital representatives.

## Results

### Demographic information of echinococcosis inpatients

Farmers (76%) were the main group of echinococcosis patients, followed by students (12.4%), workers including those self employed (5.2%), cadres (4.8%) and others (2%). Females outnumbered male patients with a ratio of 1.38. For 2000, the population ratio (0.95 females to males) [14] showed a significantly higher morbidity in females for echinococcosis (P<0.01). In south NHAR, Hui Chinese accounted for 55% of patients which matched the population composition. For north and central NHAR, 29% patients were Hui, again similar to the population composition ratio in these areas. Age at diagnosis ranged from 4–78 years (mean ± SD, 37.0±16.9) in the south, and from 6–78 years (mean ± SD, 40.0±17.0) in the north and central regions, showing a slight younger diagnosis/age in the south.

In Yinchuan, 57.7% patients came from other counties compared with the small number (28.7%) of patients from the Yinchuan area; of those who domiciled in areas other than Yinchuan, the majority (63.0%) came from central NHAR with a minority from the north (20.0%) and south (17.0%). Patients from the Guyuan hospital records were domiciled almost equally from Guayuan and from areas out of Guyuan. Among the latter, the majority (96.3%) came from various counties belonging to Guyuan prefecture in south NHAR, and were administered by the prefecture government, with a minority from north (1.0%) and central (2.7%) NHAR (Table 4).

**Table 4:** Domicile of inpatients with echinococcosis from Guyuan and Yinchuan hospitals (1996–2002).

Residence	Yinchuan* (N = 574)	Guyuan* (N = 827)
Hospital location	165 (*28.7)	378 (45.7)
Other counties in NHAR	331 (57.7)	405 (49.0)
North	65 (20.0)	4 (1.0)
Central	210 (63.4)	11 (2.7)
South	56 (17.0)	390 (96.3)
Other province	78 (13.6)	44 (5.3)

*Values in brackets are %.

### Disease and treatment information for echinococcosis inpatients

Small hydatid cysts (≤5 cm diameter) accounted for 10–12%, middle-sized cysts (5–10 cm) accounted for 50–52%, large cysts (≥10 cm) accounted for 33–37% and very large cysts (≥20 cm) accounted for 0.3–1.8% of echinococcosis patients. The majority of patients (81%) had single cysts, whereas15% had two cysts, and 4% had three or more cysts.

The duration of hospitalization pre-surgery ranged from 0–65 days (mean ± SD; 5±5 days), the number of post-surgery hospitalisation days ranged from 1–63 (mean ± SD; 10±6 days), and the average number of hospitalization days was 14±8 (mean ± SD) for all pooled records. The major reason for prolonged hospitalization prior to surgery was due to supportive treatment to improve the general health condition of the patient or for reducing the surgical risk due to other disorders, such as co-infection. Prolonged hydatid cyst drainage was the major cause of prolonged stay post-surgery. On occasions, some patients stayed for less than one day pre-surgery due to an emergency such as anaphylactic shock caused by cystic rupture which required immediate surgery.

The individual components of hospital charges were compared for echinococcosis case treatment in the rural (those county hospitals in the south) and urban (four public hospitals in Yinchuan city) hospitals (Table 5). The general expenses (hospital stay/bed fee) which accounted for 5.5% of the total for rural hospitals and 11.4% in Yinchuan, and other charges that mainly indicated costs for occupation of a hospital room by accompanying family members, or renting toilet containers for the patient, accounting for 6.4% costs in the rural areas and 3.7% in Yinchuan. The comparisons between rural and Yinchuan hospitals showed the differences were significant (P<0.05). None of the other costs showed significant differences though different percentage values were apparent when the comparisons were carried out between the rural and Yinchuan hospitals. These included the costs of surgery (36.6% in rural versus 42.1% in Yinchuan); of routine clinical laboratory tests (1.1% versus 2.2%); of medical/physical checks and pathology examinations (1.5% versus 1.8%); of provision of drugs (31.4% versus 22.0%); and of post-surgery nursing (4.1% versus 3.7%). There were also similar costs between the rural and the urban Yinchuan hospitals when a comparison was made of imaging charges for X-ray, ultrasound, and computerized tomography (CT)/magnetic resonance imaging (MRI) scanning (1.7%, 2.2% and 9.2–9.5%), respectively.

**Table 5:** A comparison of the average inpatient charges (as a %) between rural (Guyuan) and urban (Yinchuan) hospitals during 1996 to 2002.

Items	Rural (%)	Urban (%)
CT/MRI	9.5	9.2
US	2.2	2.2
X-ray	1.7	1.7
Laboratory tests	1.1	2.2
Pathology and physical examination	1.5	1.8
Surgery^1^	36.6	42.1
Drugs	31.4	22.0
Post-surgery nursing	4.1	3.7
Others items^2^	6.4	3.7
Hospital-stay/bed fee	5.5	11.4

1 Includes costs for anaesthesia, surgery and other supplemental charges.

2 Includes charges for occupation of a hospital room by accompanying family members and renting toilet containers for the patient.

### Different disease burden of human CE caused by costs for diagnosis and treatment of hospitalization in different years and locations

The different disease burden, caused by charges for hospitalization, was measured by the comparison of the different proportions of hospital-charges per capita GDP averaged between different years, between different locations and/or between different socioeconomic groups of people. A comparison (Table 1) of inpatient hospital charges for echinococcosis cases in different areas between 1998 and 2002 showed that these had increased significantly. However, a comparison of the increased ratio of local GDP with the increased ratio for the average local hospital charge for 1998 and 2002, showed they were the same for rural counties (1.3 versus 1.3) and Guyuan in the south (2.0 versus 2.0). In contrast, hospital charges increased more than the per capita GDP in Wuzhong, central NHAR in 2002 compared with 1998 (1.9 versus 1.5); for Yinchuan, the ratio of hospital charges decreased compared with the increase in GDP over this period (1.6 versus 2.3). Due to their geographic location and administrative seniority, Guyuan and Yinchuan Hospitals generally act as the centres for the health care of all people in NHAR. However, Guyuan city hospital charges were 1.3 times higher than local counties in 2002 compared with 0.9 times in 1998; in comparison, Yinchuan city hospital charge were 1.9 times higher than local county hospitals in 2002 and 1.7 times higher in 1998 (Table 1).

### Income inequalities of the different localities and socioeconomic groups can impact health care conditions and choice

A comparison of costs for echinococcosis inpatients in different hospitals in 2002 with the local income levels at the same time (Table 2) showed the local health care costs (surgery or hospitalization charges) accounted for 32% of the per capita income and 54% of the deposited income in Yinchuan in the north, but accounted for 50%, 61% and 65% per capita income and 103%, 133% and 147% deposited income in Wuzhong, Guyuan and local counties in south NHAR with low GDP, respectively. Based on the charges at various level hospitals, we calculated the costs for a poor farmer from a rural area seeking inpatient healthcare at different levels of the public hospital system. The gap between income and hospital payment accounted for 163% of per capita income and 1800% of deposited income if a poor person sought treatment at a local county hospital; it accounted for 208% of per capita income and 2285% of deposited income if sought in Guyuan (defined as a rural area), and 318% of per capita income and 3495% of deposited income if sought in Yinchuan (defined as an urban area) (Table 2).

## Discussion

Although average incomes have risen recently in south NHAR, health care costs have also increased considerably. The rise in the average cost of an admission to a county hospital in south NHAR was similar to the growth in average income, while the average cost of admission in Yinchuan, the capital city of NHAR, in the north, has become relatively more expensive. Evidence for increased disease burden, particularly for rural patients, was demonstrated by the fact that the average hospital charge in Yinchuan was 1.7 times higher than that for a rural county hospital in 1998 and this rate increased to 1.9 times in 2002; the rate of charge differences for Guyuan versus rural county hospitals also increased from 0.85 to 1.26 times (1998–2002) (Table 1). Therefore, the first choice for clinical consultation and hospitalization for most of the rural population in NHAR is the local county hospital because of its closer proximity and the lower health care costs charged.

This study has provided clear evidence that the cost of hospitalization imposes a severe burden on many rural households, particularly poorer families, in NHAR. Households had to spend a large proportion of their annual income on medical costs. Substantial numbers of rural households had to borrow money to pay hospital fees, because the payments were greater than their annual income. This situation is similar to other settings [15] where the most common reason for not seeking hospital inpatient treatment is financial hardship. Such financial problems restrict poor people access to quality health care. The most severe effects are on those who did not seek hospital treatment because of their inability to afford treatment and consequently their sickness goes untreated. Such people are at risk of further suffering and deterioration in health [16], [19]. In addition, some patients may have limited access to professional health care services, poor access to drugs, are only able to purchase drugs from cheaper sources, or seek treatment at cheaper private clinics where the health care facilities generally are limited [20]. This situation not only generates an unhealthy, irrational use of drugs, but also wastes scarce financial resources. Due to the financial constraints, poor people delay seeking care until an emergency situation arises, but this delay often forces them to seek care at a more expensive level, typically in hospitals. This may also be a major reason for our previous report of hospitalized patients with echinococcosis having more severe disease [16]. There are two main reasons why only a low percentage (17.0%) of patients from poor rural areas in south NHAR attends hospitals in Yinchuan. They may either be unwilling to pay transportation charges or the more expensive hospital fees compared with those of the local county hospitals are prohibitive. The facilities in the county hospitals are generally of poorer quality and diagnostic procedures and treatment are less efficient than in urban hospitals. Household income levels strongly influenced the choice of hospital and the additional expense impeded access of poor people to better quality health care.

Hospitals in rural P.R. China rarely provide non-medical care (e.g. food, drinking water, toilet facilities) for inpatients who are cared for by their relatives, generally farm labourers. In addition, a sick household member is unable to work or do heavy tasks for long periods of time; prolonged illness and recuperation can therefore influence production and income for one or more production cycle (eg. annual/seasonal harvest), which increases the long-term financial burden on the household.

Rural families are faced with a range of disease problems, but the need for medical help clearly exceeds their ability to pay for treatment. This inevitably results in poor livelihood and financial difficulty. People use a variety of ways to finance unforeseen health expenditure; they use savings or sell consumables, borrow money or purchase health care on credit, often leaving them with substantial debt [19]. The families of many patients cut down on food to offset the cost of borrowing, even sacrificing investment in future productivity, such as withdrawal of their children from school to support the family through labour, and to save school fees [20], [21]. This inevitably triggers a vicious circle of impoverishment and causes more debt [22] because these poorly educated children will obviously be part of low-income socio-status groups in the future. The high cost that poor rural people have to pay to health providers for treatment is an unfortunate path that leads this group from illness to poverty.

Economically vulnerable people have little capita assets or savings to withstand any short-term reduction in income without falling quickly into poverty. So, any disaster, such as illness or an accident, requiring time off work, may bring about a financial crisis leading to a rapid deterioration in livelihood. Therefore, poverty resulting from illness, due especially to human echinococcosis, has become a significant social problem in rural NHAR [10].

The costs for a hospital bed and the period spent in hospital for hydatid surgery and treatment were higher in urban than rural hospitals in NHAR. Reduction in these costs by hospital management would allow the limited financial resources to be used for diagnosis and treatment of cases. As well, the socioeconomic problems arising from echinococcosis which add to the burden on patients' families and the community were not taken into account in the current calculations. This additional information needs to be collected as a pre-requisite for developing more comprehensive government policies to tackle the burden of echinococcosis in rural NHAR. We advocate that the local and central Chinese governments should increase investment in health care generally in poor rural areas, and launch relevant medical aid projects to help those in poverty, and improving the equity of the health care system. In order to improve access of rural residents to health care services, it is important to locate health facilities and personnel rationally, thereby reducing the financial commitment and distance as obstacles to better health care.

Hospital costs for each hydatid disease surgical case were the best assessment of the average expenditure per patient for NHAR hospitals although the total illness burden costs will be underestimated. This study has some limitations in that it is hospital-based, in which other health care costs, such as consultation fees, transport, food costs, and the convalescence, rehabilitation, and the social consequences of disability have not been included. In addition, since this set of data was obtained from retrospective hospital records, the economic status of each patient could not be obtained. Therefore, the financial burden analysis was based on comparisons between the average of the aggregate patient group charges and the average local level of per capita income. Such aggregated correlations only reflect information at the group level, and do not target an individual patient. However, taking a population level perspective into account, this study provides important information for future policy development to tackle the public health issues for this rural population, and elsewhere, where the situation may be comparable to that in rural P.R. China.
